# Supplementary material for: Microsaccadic eye movement generation is sufficient to enhance peripheral visual sensitivity
Source: PLoS Biol. 2026 Aug 21;24(8):e3003968. doi: 10.1371/journal.pbio.3003968 (PMC13521465; doi:10.1371/journal.pbio.3003968)
Supplement: Supplementary file 1 [file pbio.3003968.s002.pdf]

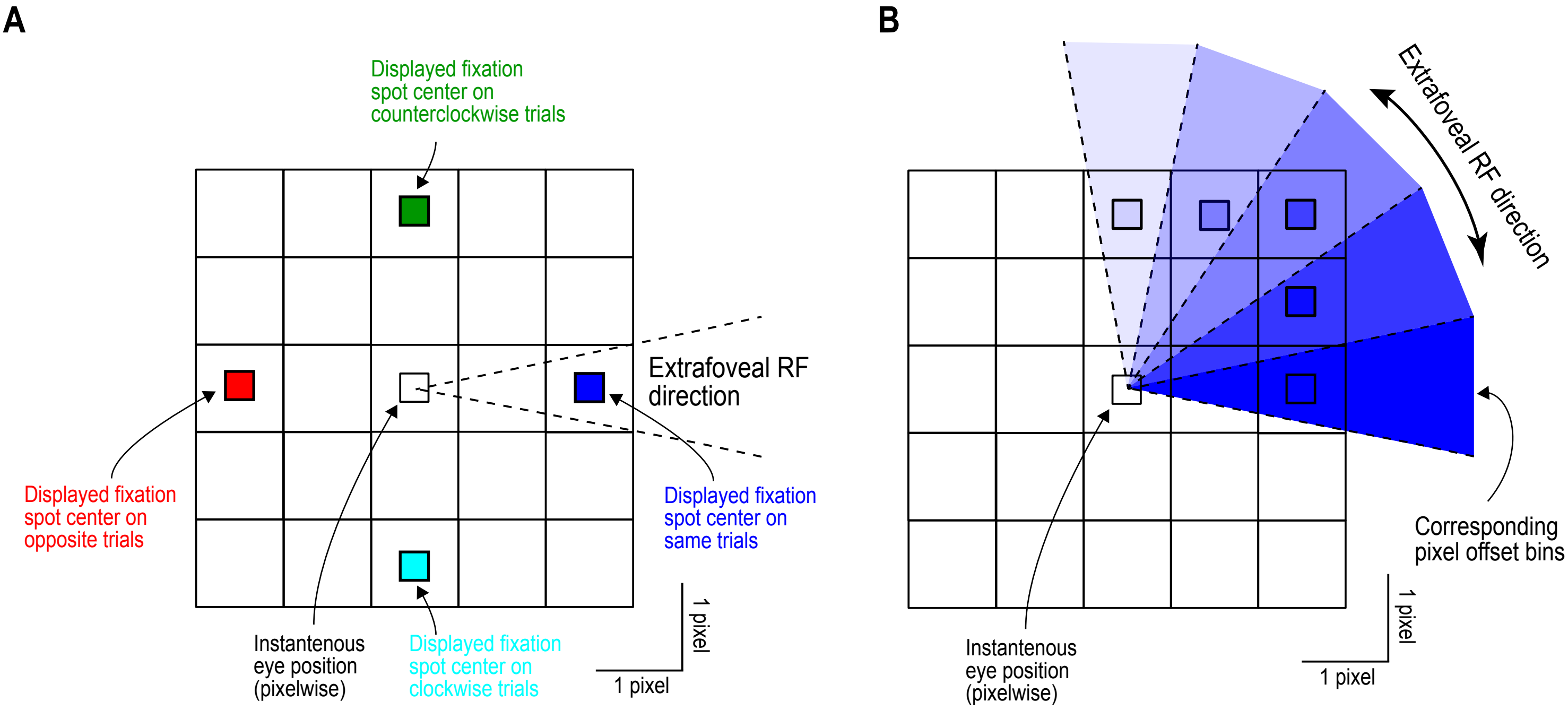
A
Displayed fixation spot center on counterclockwise trials
Extrafoveal RF direction
Displayed fixation spot center on opposite trials
Displayed fixation spot center on same trials
Instantenous eye position (pixelwise)
Displayed fixation spot center on clockwise trials
1 pixel
1 pixel
B
Extrafoveal RF direction
Corresponding pixel offset bins
Instantenous eye position (pixelwise)
1 pixel
1 pixel
