# Supplementary material for: Microsaccadic eye movement generation is sufficient to enhance peripheral visual sensitivity
Source: PLoS Biol. 2026 Aug 21;24(8):e3003968. doi: 10.1371/journal.pbio.3003968 (PMC13521465; doi:10.1371/journal.pbio.3003968)
Supplement: Supplementary file 2 [file pbio.3003968.s003.pdf]

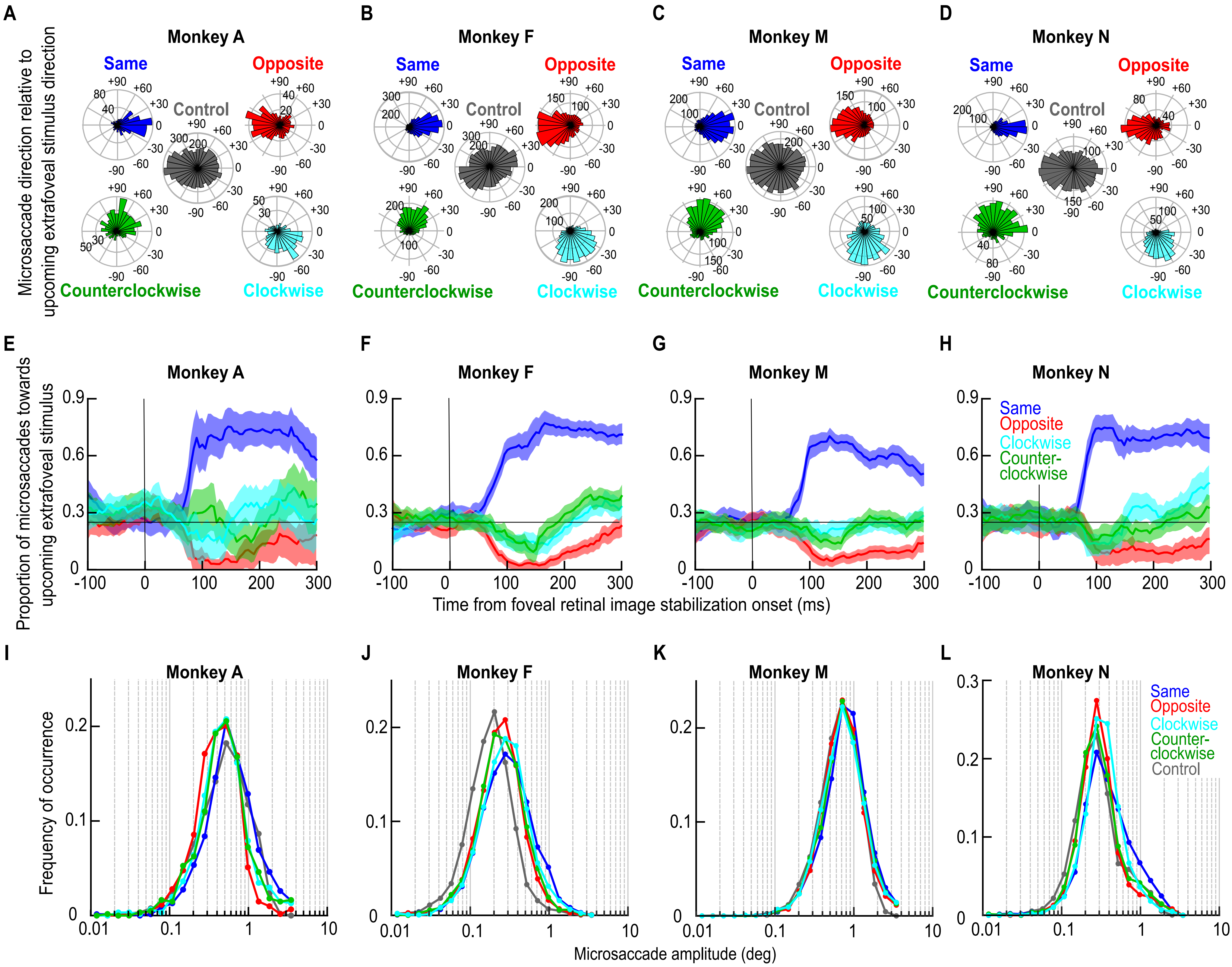
A
B
C
D
Monkey A
Monkey F
Monkey M
Monkey N
Same
Opposite
Control
Counterclockwise
Clockwise
Microsaccade direction relative to upcoming extrafoveal stimulus direction
E
F
G
H
Proportion of microsaccades towards upcoming extrafoveal stimulus
Time from foveal retinal image stabilization onset (ms)
Same
Opposite
Clockwise
Counter-clockwise
I
J
K
L
Frequency of occurrence
Microsaccade amplitude (deg)
Same
Opposite
Clockwise
Counter-clockwise
Control
