# Supplementary material for: Microsaccadic eye movement generation is sufficient to enhance peripheral visual sensitivity
Source: PLoS Biol. 2026 Aug 21;24(8):e3003968. doi: 10.1371/journal.pbio.3003968 (PMC13521465; doi:10.1371/journal.pbio.3003968)
Supplement: Supplementary file 3 [file pbio.3003968.s004.pdf]

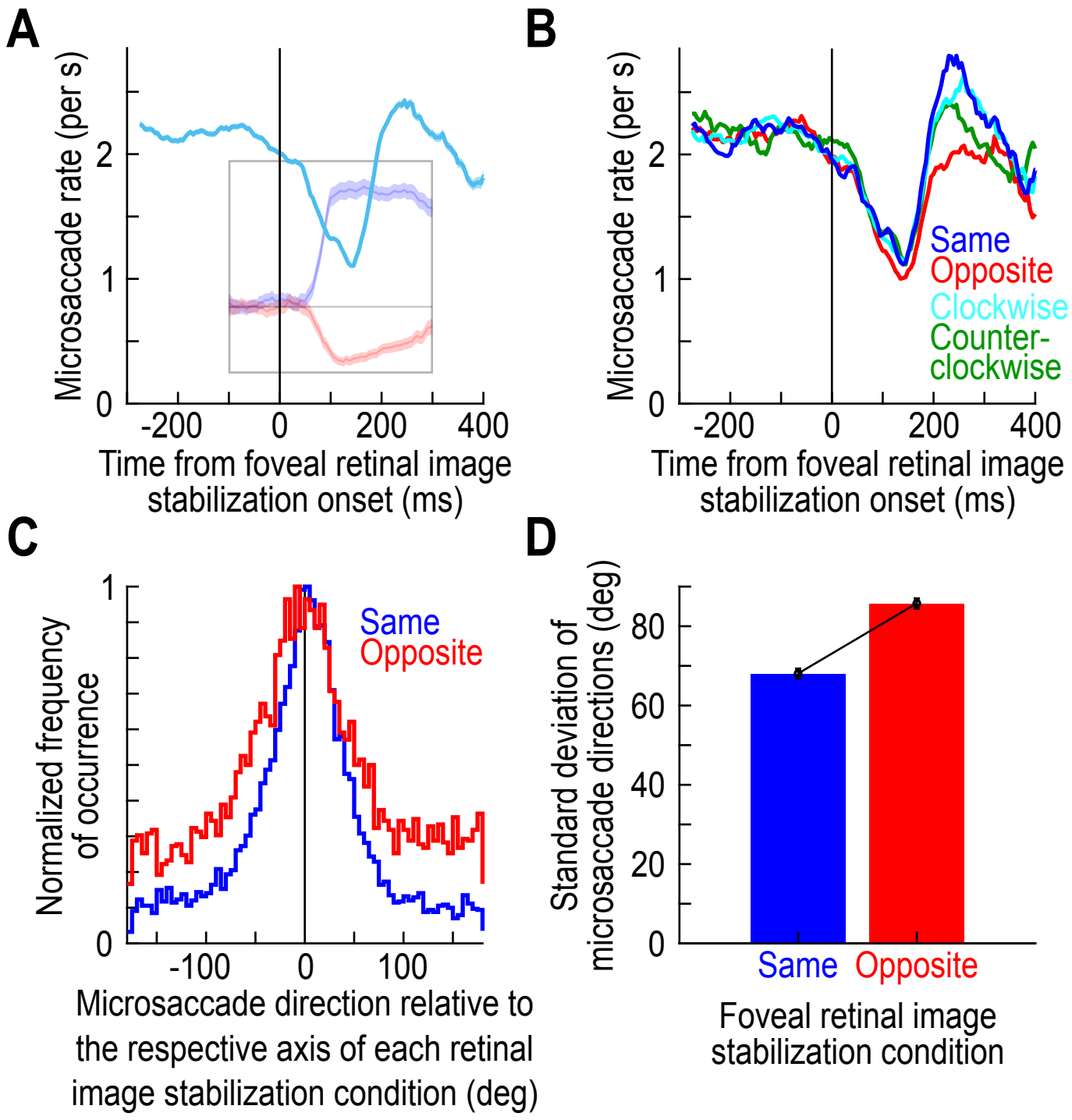
A
Microsaccade rate (per s)
Time from foveal retinal image stabilization onset (ms)
B
Microsaccade rate (per s)
Same
Opposite
Clockwise
Counter-clockwise
Time from foveal retinal image stabilization onset (ms)
C
Normalized frequency of occurrence
Same
Opposite
Microsaccade direction relative to the respective axis of each retinal image stabilization condition (deg)
D
Standard deviation of microsaccade directions (deg)
Same
Opposite
Foveal retinal image stabilization condition
