# Supplementary material for: Microsaccadic eye movement generation is sufficient to enhance peripheral visual sensitivity
Source: PLoS Biol. 2026 Aug 21;24(8):e3003968. doi: 10.1371/journal.pbio.3003968 (PMC13521465; doi:10.1371/journal.pbio.3003968)
Supplement: Supplementary file 4 [file pbio.3003968.s005.pdf]

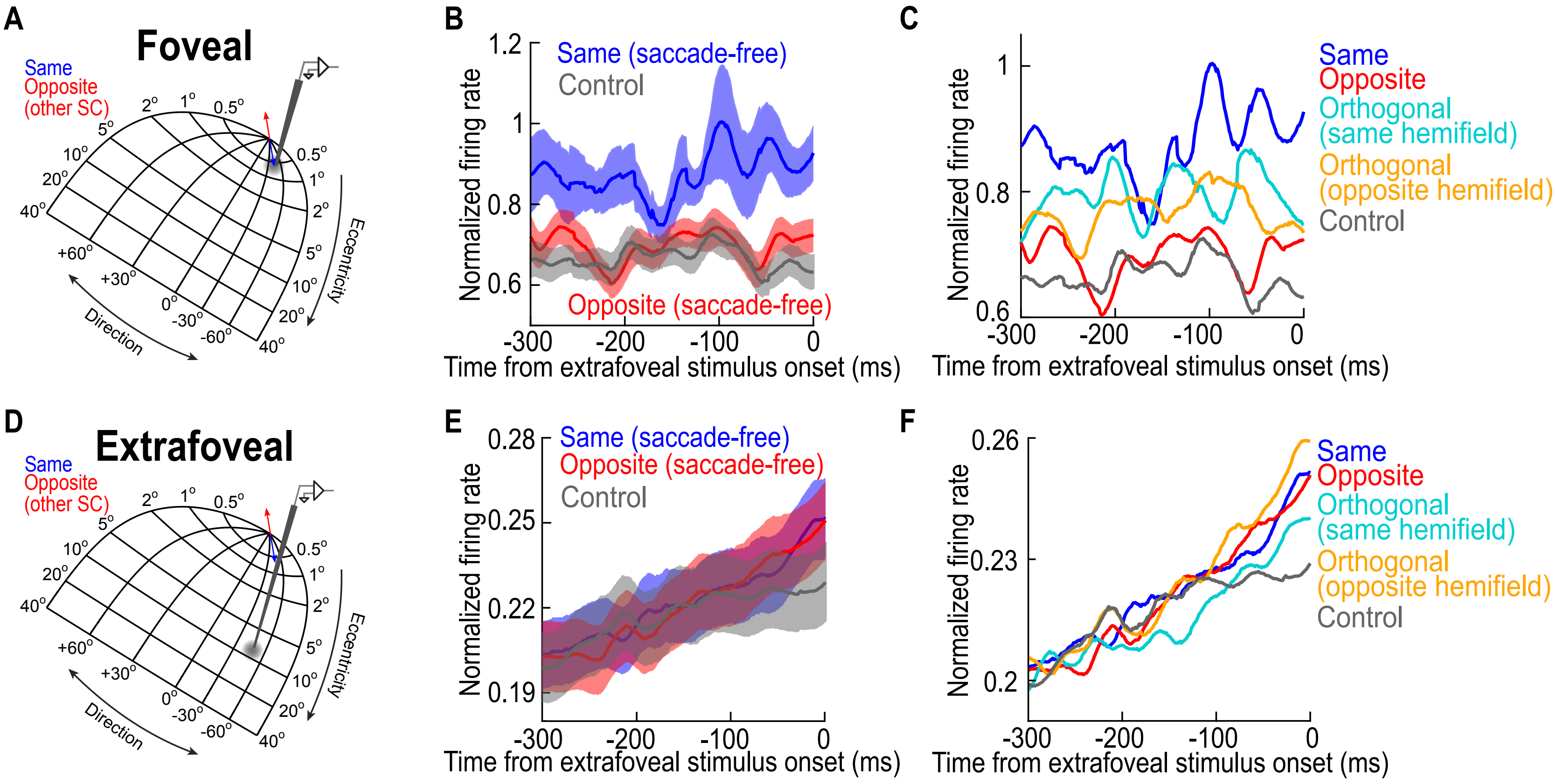
A
Foveal
Same
Opposite
(other SC)
Direction
Eccentricity
B
Same (saccade-free)
Control
Opposite (saccade-free)
Normalized firing rate
Time from extrafoveal stimulus onset (ms)
C
Same
Opposite
Orthogonal
(same hemifield)
Orthogonal
(opposite hemifield)
Control
Normalized firing rate
Time from extrafoveal stimulus onset (ms)
D
Extrafoveal
Same
Opposite
(other SC)
Direction
Eccentricity
E
Same (saccade-free)
Opposite (saccade-free)
Control
Normalized firing rate
Time from extrafoveal stimulus onset (ms)
F
Same
Opposite
Orthogonal
(same hemifield)
Orthogonal
(opposite hemifield)
Control
Normalized firing rate
Time from extrafoveal stimulus onset (ms)
