# Supplementary material for: Microsaccadic eye movement generation is sufficient to enhance peripheral visual sensitivity
Source: PLoS Biol. 2026 Aug 21;24(8):e3003968. doi: 10.1371/journal.pbio.3003968 (PMC13521465; doi:10.1371/journal.pbio.3003968)
Supplement: Supplementary file 5 [file pbio.3003968.s006.pdf]

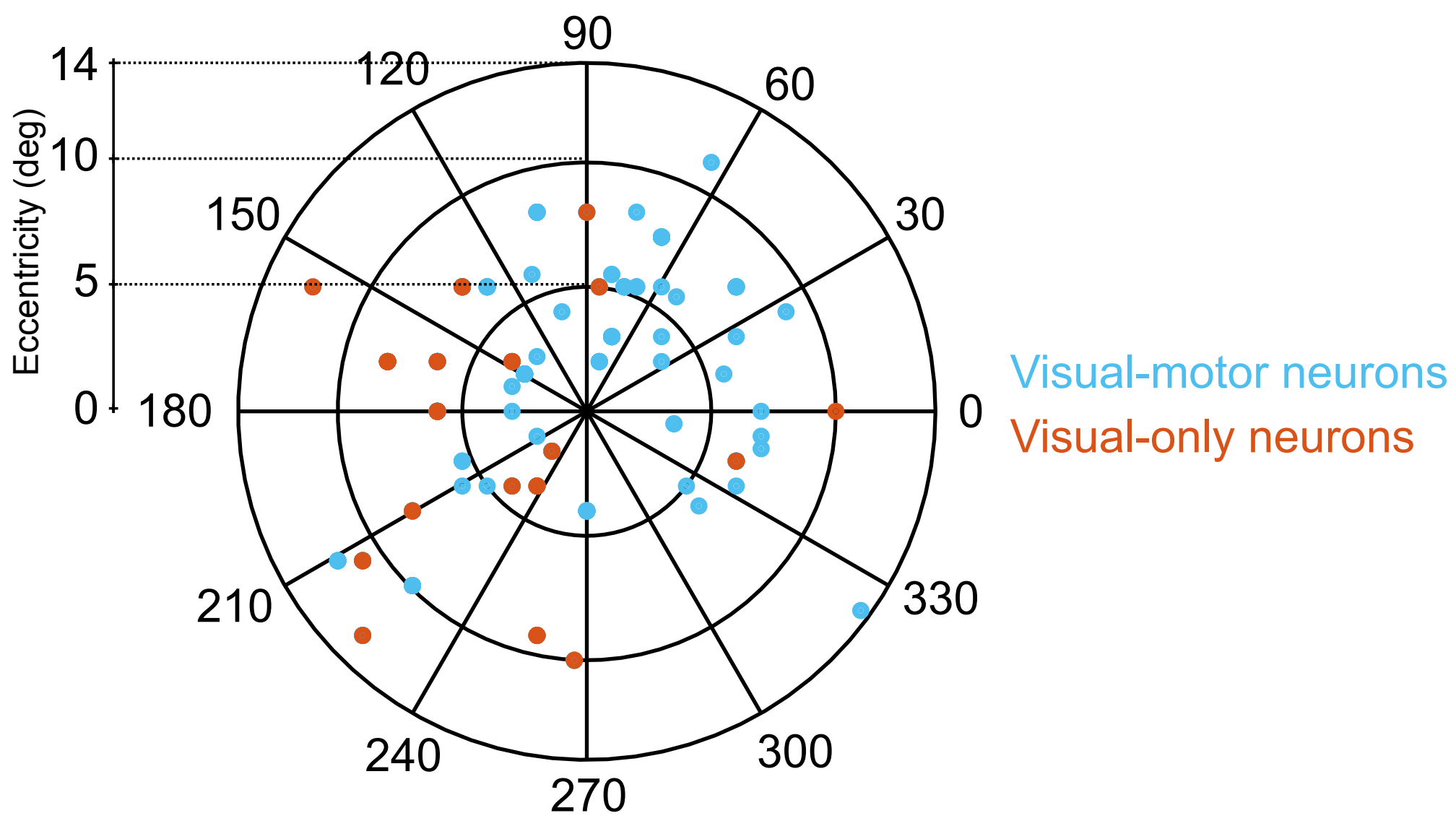
Eccentricity (deg)
14
10
5
0
90
60
120
30
150
180
0
210
330
240
300
270
Visual-motor neurons
Visual-only neurons
