# Supplementary material for: Microsaccadic eye movement generation is sufficient to enhance peripheral visual sensitivity
Source: PLoS Biol. 2026 Aug 21;24(8):e3003968. doi: 10.1371/journal.pbio.3003968 (PMC13521465; doi:10.1371/journal.pbio.3003968)
Supplement: Supplementary file 6 [file pbio.3003968.s007.pdf]

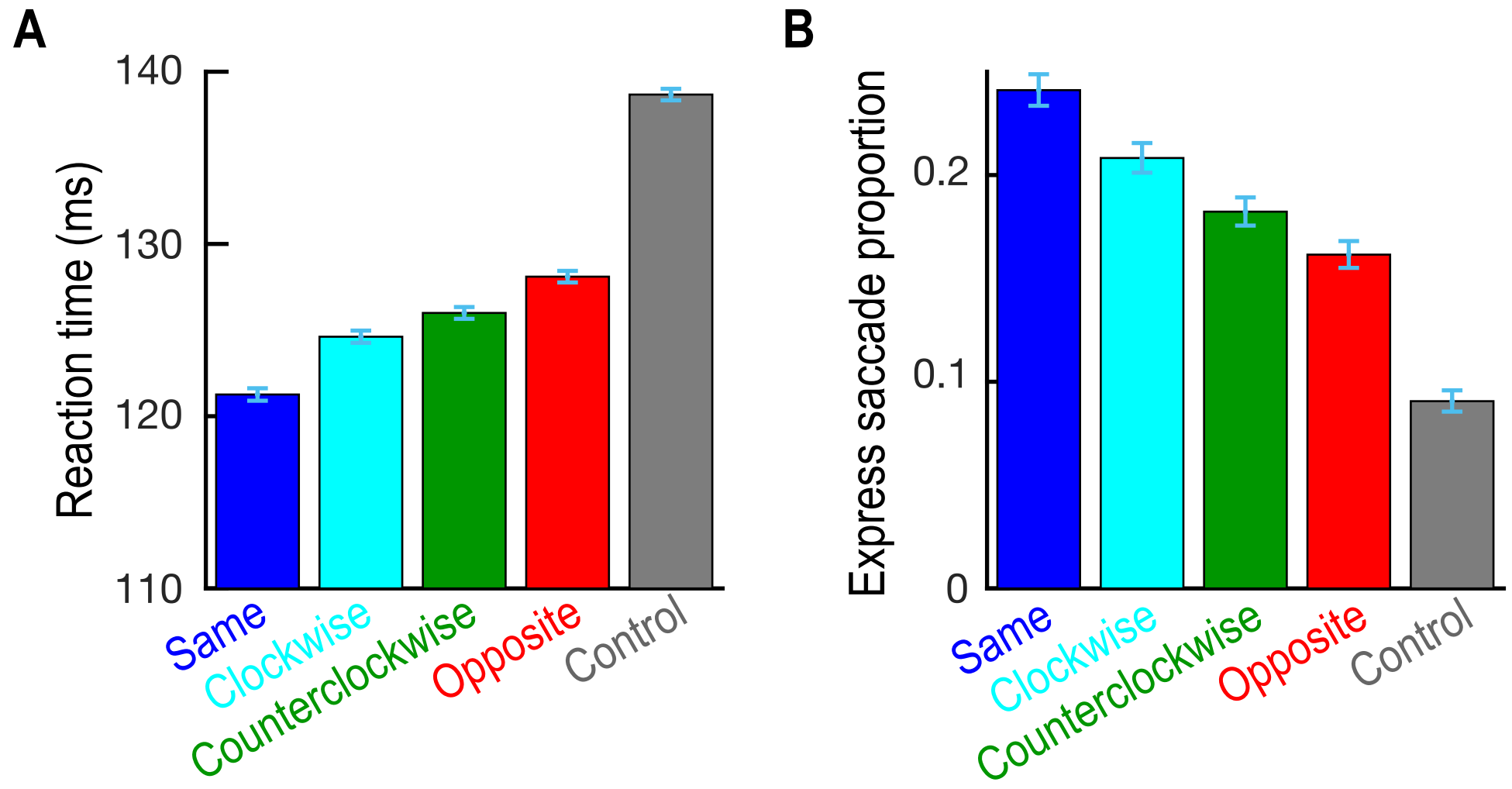
A
140
130
120
110
Reaction time (ms)
Same
Clockwise
Counterclockwise
Opposite
Control
B
0.2
0.1
0
Express saccade proportion
Same
Clockwise
Counterclockwise
Opposite
Control
