# Supplementary material for: Microsaccadic eye movement generation is sufficient to enhance peripheral visual sensitivity
Source: PLoS Biol. 2026 Aug 21;24(8):e3003968. doi: 10.1371/journal.pbio.3003968 (PMC13521465; doi:10.1371/journal.pbio.3003968)
Supplement: Supplementary file 7 [file pbio.3003968.s008.pdf]

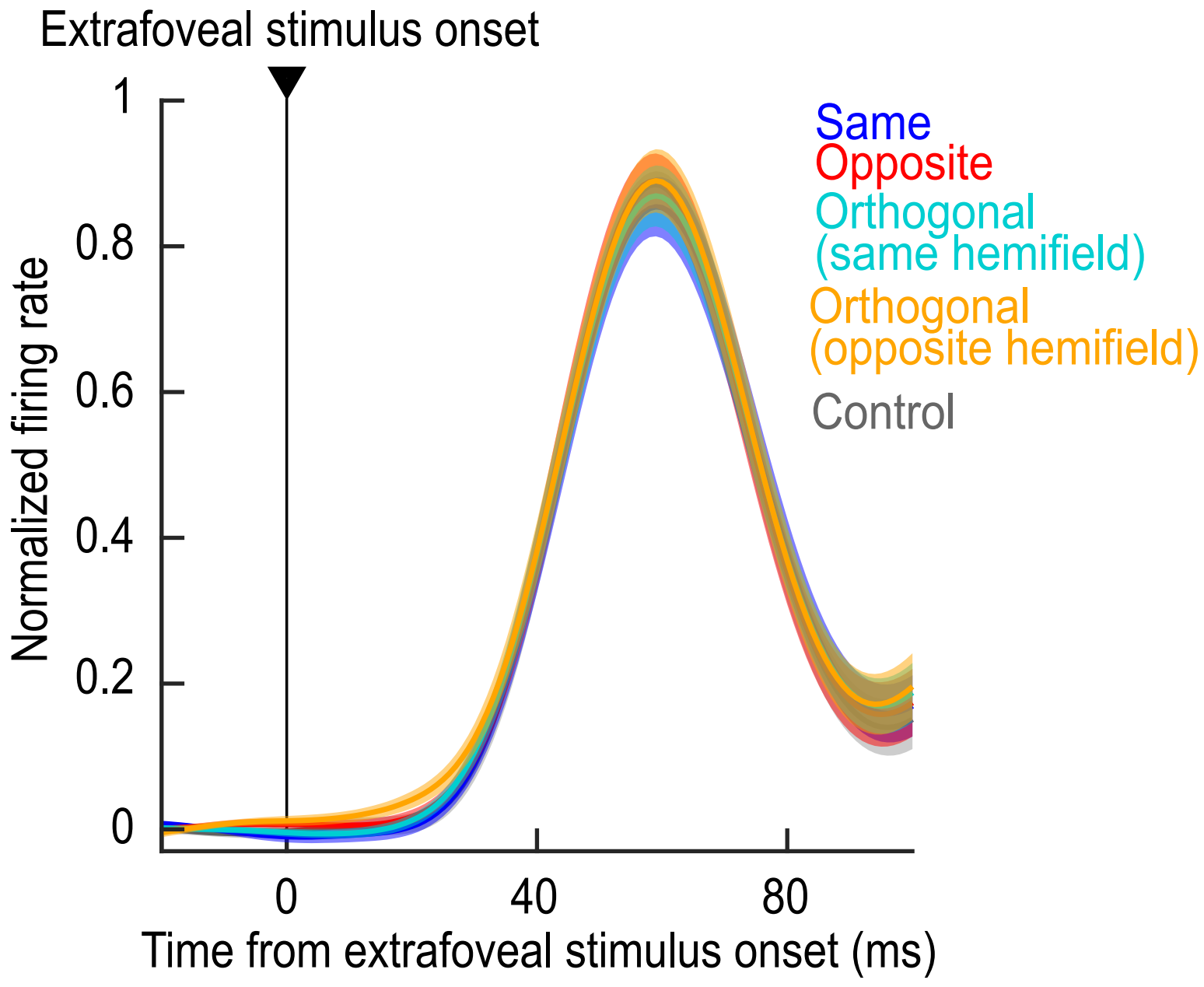

Extrafoveal stimulus onset
Normalized firing rate
1
0.8
0.6
0.4
0.2
0
0
40
80
Time from extrafoveal stimulus onset (ms)
Same
Opposite
Orthogonal
(same hemifield)
Orthogonal
(opposite hemifield)
Control
