# Supplementary material for: Microsaccadic eye movement generation is sufficient to enhance peripheral visual sensitivity
Source: PLoS Biol. 2026 Aug 21;24(8):e3003968. doi: 10.1371/journal.pbio.3003968 (PMC13521465; doi:10.1371/journal.pbio.3003968)
Supplement: Supplementary file 8 [file pbio.3003968.s009.pdf]

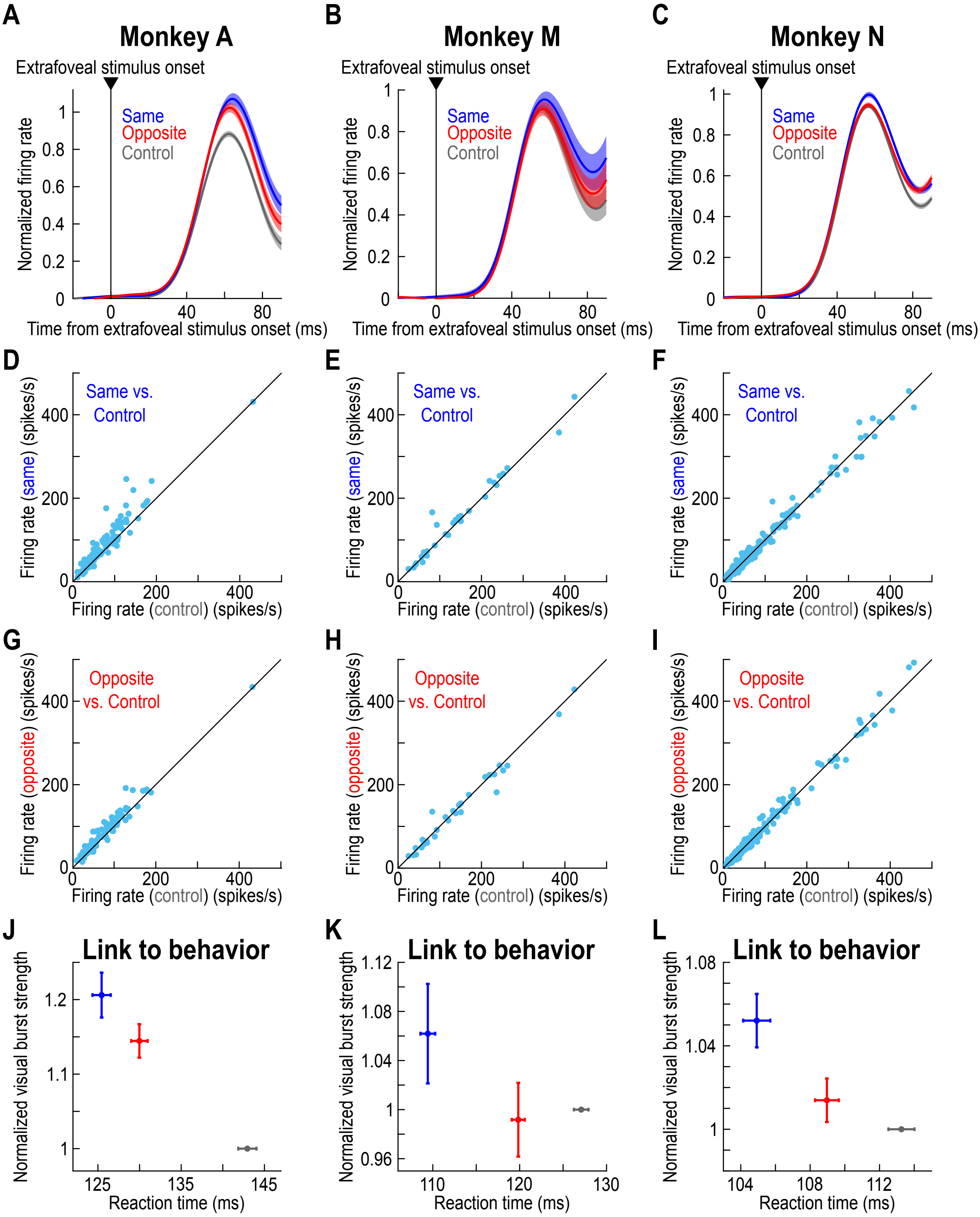
A
Monkey A
Extrafoveal stimulus onset
Same
Opposite
Control
Normalized firing rate
Time from extrafoveal stimulus onset (ms)
B
Monkey M
C
Monkey N
D
Same vs. Control
Firing rate (same) (spikes/s)
Firing rate (control) (spikes/s)
E
F
G
Opposite vs. Control
Firing rate (opposite) (spikes/s)
H
I
J
Link to behavior
Normalized visual burst strength
Reaction time (ms)
K
L
