# Supplementary material for: Microsaccadic eye movement generation is sufficient to enhance peripheral visual sensitivity
Source: PLoS Biol. 2026 Aug 21;24(8):e3003968. doi: 10.1371/journal.pbio.3003968 (PMC13521465; doi:10.1371/journal.pbio.3003968)
Supplement: Supplementary file 9 [file pbio.3003968.s010.pdf]

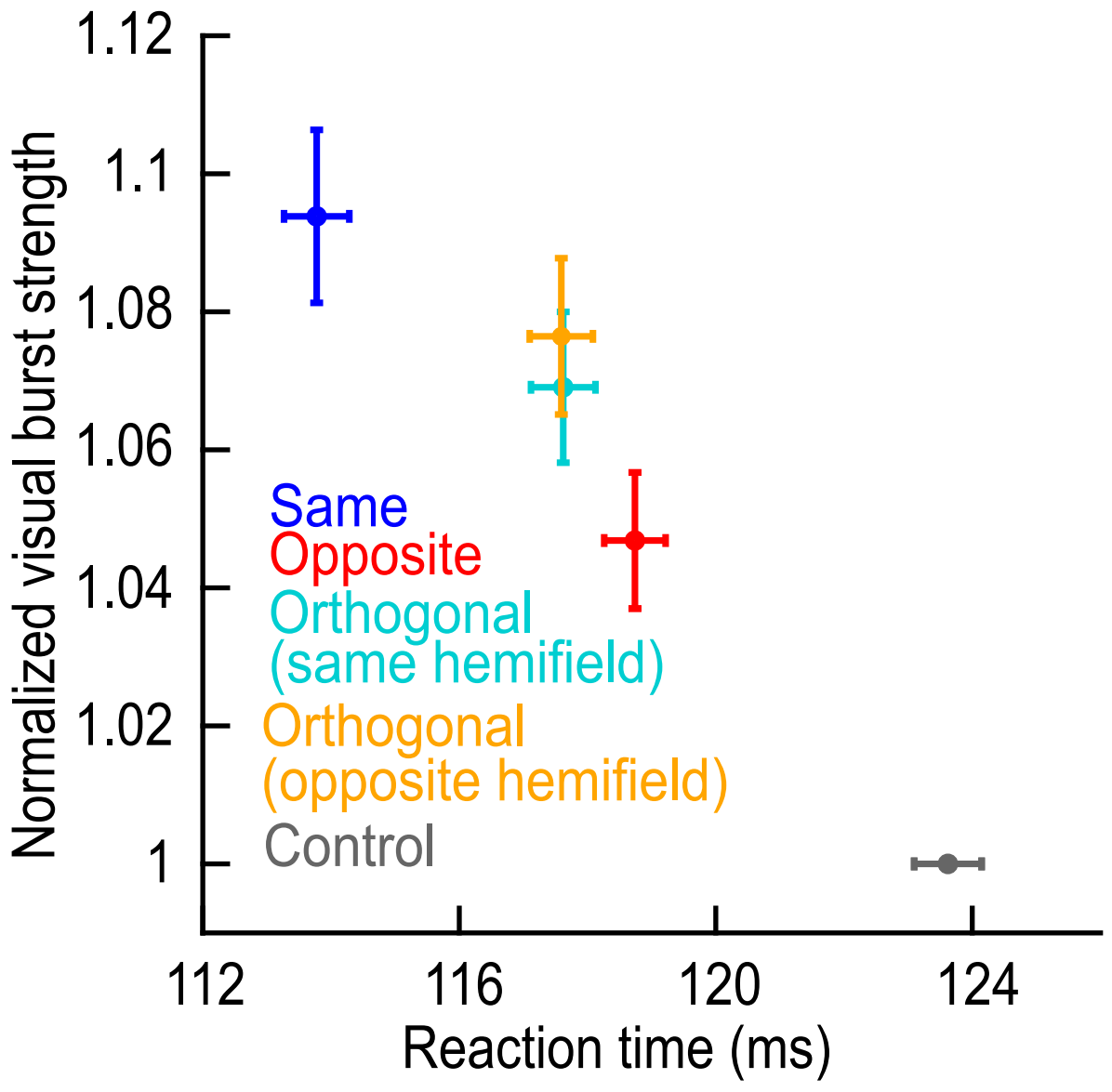

1.12
1.1
1.08
1.06
1.04
1.02
1
Normalized visual burst strength
Same
Opposite
Orthogonal
(same hemifield)
Orthogonal
(opposite hemifield)
Control
112
116
120
124
Reaction time (ms)
