# Supplementary material for: Microsaccadic eye movement generation is sufficient to enhance peripheral visual sensitivity
Source: PLoS Biol. 2026 Aug 21;24(8):e3003968. doi: 10.1371/journal.pbio.3003968 (PMC13521465; doi:10.1371/journal.pbio.3003968)
Supplement: Supplementary file 10 [file pbio.3003968.s011.pdf]

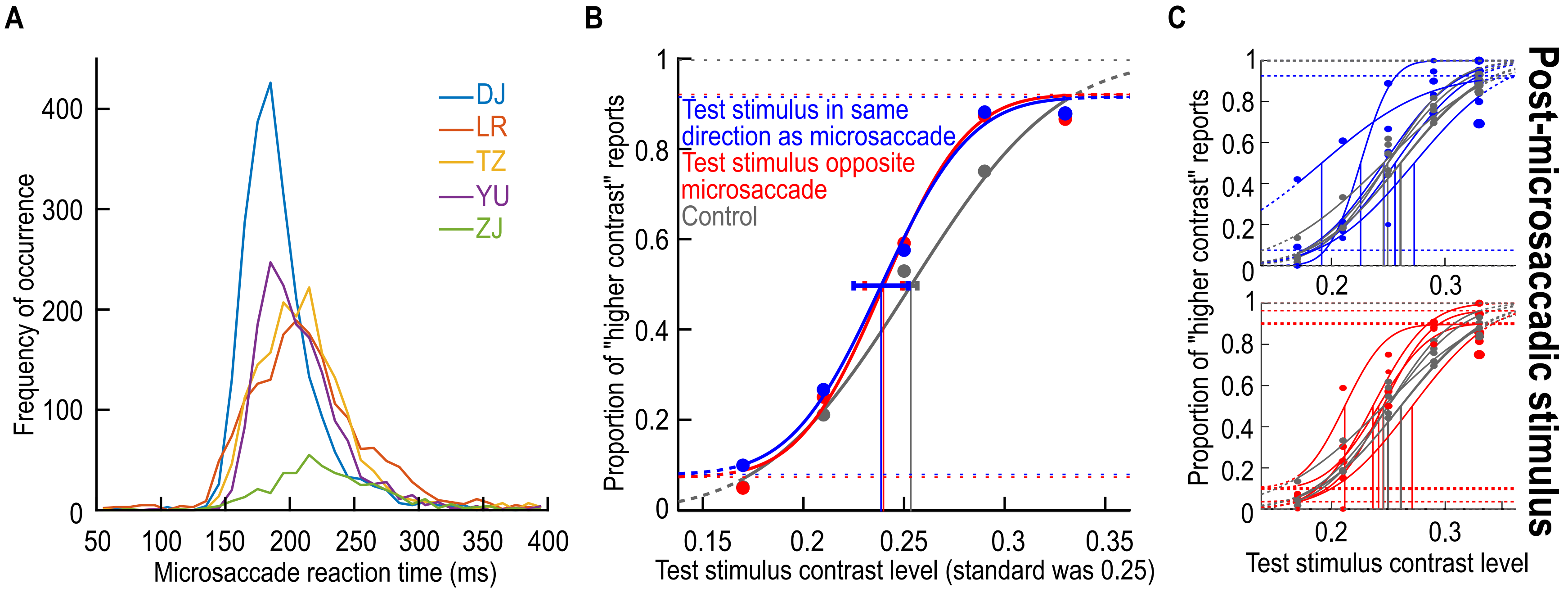

A
Frequency of occurrence
Microsaccade reaction time (ms)
DJ
LR
TZ
YU
ZJ
B
Proportion of "higher contrast" reports
Test stimulus in same direction as microsaccade
Test stimulus opposite microsaccade
Control
Test stimulus contrast level (standard was 0.25)
C
Post-microsaccadic stimulus
Proportion of "higher contrast" reports
Test stimulus contrast level
