# Supplementary material for: Microsaccadic eye movement generation is sufficient to enhance peripheral visual sensitivity
Source: PLoS Biol. 2026 Aug 21;24(8):e3003968. doi: 10.1371/journal.pbio.3003968 (PMC13521465; doi:10.1371/journal.pbio.3003968)
Supplement: Supplementary file 11 [file pbio.3003968.s012.pdf]

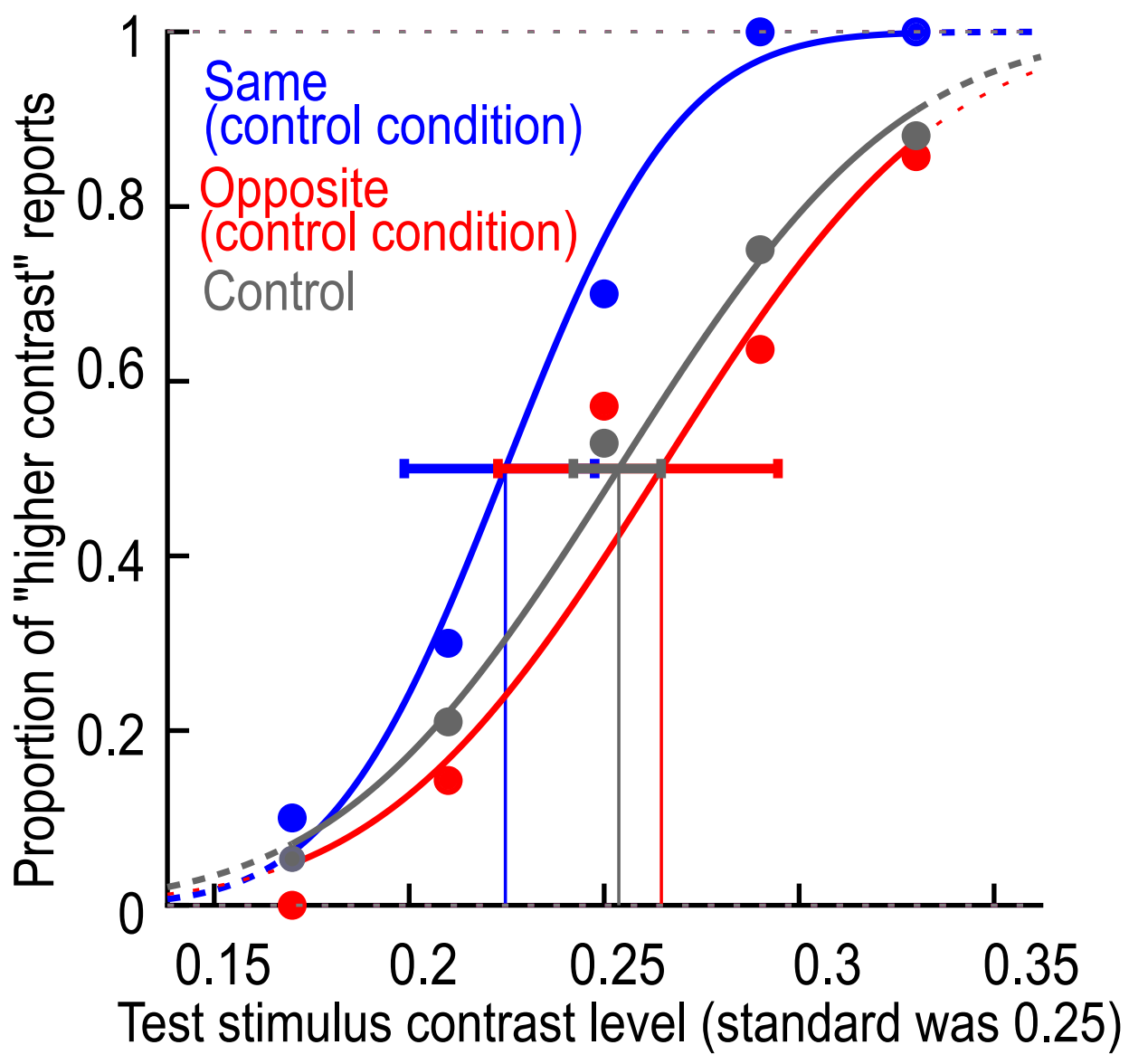

Same
(control condition)
Opposite
(control condition)
Control
Proportion of "higher contrast" reports
1
0.8
0.6
0.4
0.2
0
0.15
0.2
0.25
0.3
0.35
Test stimulus contrast level (standard was 0.25)
