# Supplementary material for: Microsaccadic eye movement generation is sufficient to enhance peripheral visual sensitivity
Source: PLoS Biol. 2026 Aug 21;24(8):e3003968. doi: 10.1371/journal.pbio.3003968 (PMC13521465; doi:10.1371/journal.pbio.3003968)
Supplement: Supplementary file 12 [file pbio.3003968.s013.pdf]

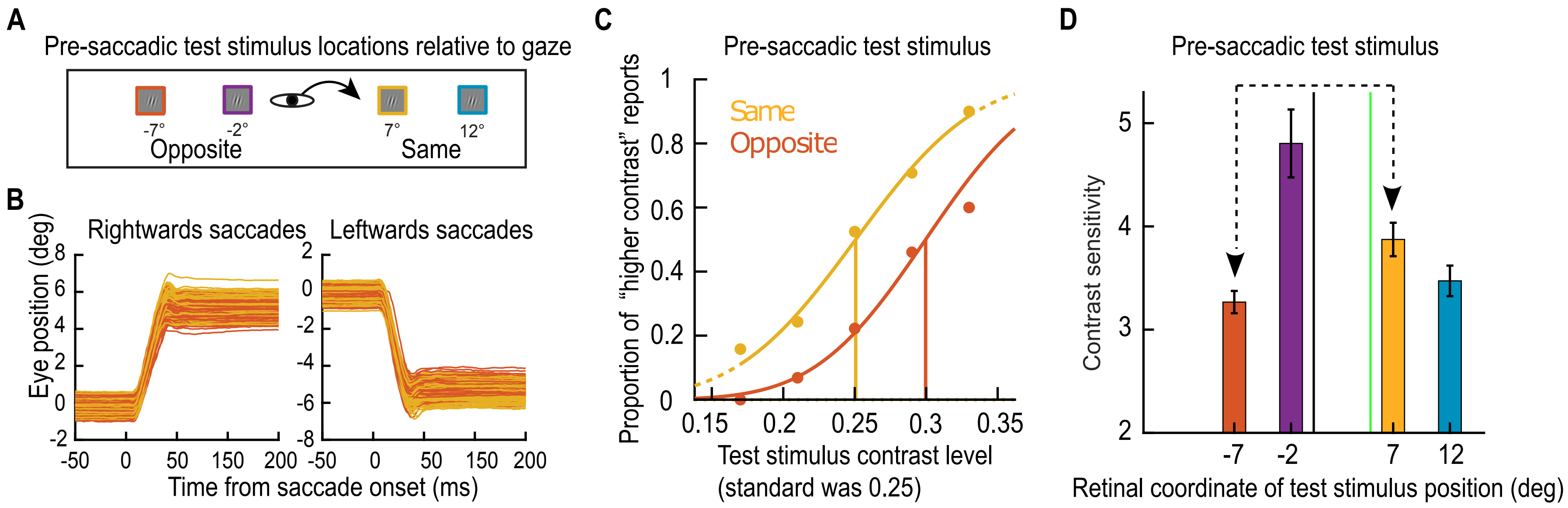
A
Pre-saccadic test stimulus locations relative to gaze
-7°
-2°
Opposite
7°
12°
Same
B
Rightwards saccades
Leftwards saccades
Eye position (deg)
Time from saccade onset (ms)
C
Pre-saccadic test stimulus
Same
Opposite
Proportion of "higher contrast" reports
Test stimulus contrast level
(standard was 0.25)
D
Pre-saccadic test stimulus
Contrast sensitivity
Retinal coordinate of test stimulus position (deg)
