# Supplementary material for: Microsaccadic eye movement generation is sufficient to enhance peripheral visual sensitivity
Source: PLoS Biol. 2026 Aug 21;24(8):e3003968. doi: 10.1371/journal.pbio.3003968 (PMC13521465; doi:10.1371/journal.pbio.3003968)
Supplement: Supplementary file 14 [file pbio.3003968.s016.pdf]

Responses to Reviewer Comments on:
"Foveal action for the control of extrafoveal vision"
PBIOLOGY-D-26-00289R1
June 23, 2026

We thank the editors and reviewers very much for the opportunity to publish our work in this journal.

We have now revised the manuscript in accordance with all of the reviewer comments and recommendations. We have also formatted the paper according to the journal's instructions and technical requirements.

We believe that the paper is now much improved after the revisions, and we thank the reviewers once again for their feedback.

Responses to Reviewer 1

Reviewer #1: Summary of the Manuscript:
This study employs a novel Retinal Image Stabilization (RIS) technique in macaques to manipulate foveal motor planning, concurrently with neurophysiological recordings in the Superior Colliculus (SC), and combines this approach with human psychophysical experiments. The goal is to dissect the causal relationship between microsaccade generation and peripheral visual sensitivity. The authors report that the foveal oculomotor state is sufficient to causally modulate peripheral visual sensitivity and behavioral reaction times in a directionally specific manner (i.e., enhancement in the "Same" direction). Furthermore, the findings suggest this modulation is mediated by an amplification of pre-microsaccadic gain and an attenuation of post-microsaccadic suppression.
While the topic is novel and the combined RIS-electrophysiology methodology is highly innovative—offering a unique perspective on the long-standing debate regarding causality between microsaccades and attention—some concerns remain regarding the internal validity of the experimental design, particularly within the human experiments, and the control of confounding variables introduced by the RIS manipulation, such as cognitive conflict and arousal dynamics.

Thank you very much for the careful and insightful comments on our manuscript. We have made a genuine effort to fully address all of the reviewer and editor comments in the revised version.

Major Comments:

1. The authors acknowledge two critical side effects of the RIS paradigm. First, the "fighting the drift" phenomenon, in which monkeys actively counteract the retinal drift error to maintain fixation. This implies that, particularly in the "Opposite" condition, the animals likely experience a state of elevated cognitive load, characterized by a conflict between the internally generated "motor plan" and the external "task goal" of

maintaining fixation. Second, the "temporal cueing" effect, whereby the onset of the RIS itself acts as an unspecific warning signal. This latter effect explains why reaction times were slowest in the Control condition, where no directional plan is introduced.

These facts collectively suggest that the RIS manipulation does not merely isolate the "oculomotor plan" in a pure form but likely induces significant changes in the animal's global cognitive and physiological state. Consequently, the psychological and physiological contexts of the "Same," "Opposite," and "Control" conditions are not equivalent. Recent frameworks in neurodynamics (e.g., Arousal as a universal embedding for spatiotemporal brain dynamics, Nature, 2025) suggest that arousal operates not as a simple linear gain but across a complex, high-dimensional manifold. If the RIS manipulation places the "Same" and "Opposite" conditions at distinct coordinates on this arousal/cognitive state manifold, then the observed differences in visual sensitivity could be a downstream consequence of these global state alterations, rather than a direct, specific causal result of the microsaccade plan per se.

We agree that arousal did play some role in our results, and this is seen in the fact that saccadic reaction times were rendered faster than control even in the opposite trials. Also, the pre-stimulus peripheral activity rose slightly in anticipation of peripheral stimulus onset (Fig. 2F).

However, the key goal of our manipulation was to achieve a differential pre-stimulus state modulation exclusively in the foveal representation of the SC, which generates microsaccades, without having a similar such differential pre-stimulus state modulation in the periphery, where the visual bursts were to eventually occur. This was achieved with our retinal image stabilization approach, as seen in Fig. 2. Thus, even if they mapped onto different parts of an arousal manifold, our “Same” and “Opposite” manipulations still allowed us to experimentally control the state of “microsaccade motor plans” in the rostral portion of the SC, without differentially altering pre-stimulus peripheral state. We have now clarified potential links to arousal in the revised manuscript (e.g. please see **lines 340-343, 576-579, 606-609, 722-723, 1596-1599**).

Please also note that from a behavioral perspective, there was no reason to expect that the initiation of the retinal image stabilization process in the opposite trials would give rise to different arousal levels from the initiation of the process in the same trials. In fact, in the **newly added S3A Figure**, we found that microsaccadic inhibition (a hallmark of the oculomotor system detecting the transients associated with the onset of retinal image stabilization of the fixation spot) had the same timing, suggesting that both events initiated similar oculomotor responses (this newly added figure is discussed below in the context of a related comment). Thus, the differential modulation that we saw in the foveal SC (Fig. 2A-D) was the biggest differential modulation in our experiments. Consistent with this, there were no differences in microsaccade-related discharges in the different conditions (Fig. 2D). More importantly, when we analyzed peri-microsaccadic modulations in the control condition (Fig. 5), we found that the results of our retinal image stabilization were congruent with what regular microsaccades did even without any retinal image stabilization, which we find quite remarkable (please also see similar sentiments below by Reviewer 2). Importantly, some of these peri-microsaccadic modulations in Fig. 5 actually reduced visual sensitivity, rather than increased it (in the post-

microsaccadic interval particularly); this is not easily explained by a global arousal or warning signal.

Finally, we note that the oculomotor system has to constantly fight drift in the control condition as well, and not just in retinal image stabilization. The difference is merely that our experimental manipulation amplified this by virtue of the constant forced error for a prolonged period of time (please see **lines 197-201**).

As mentioned above, all of these points were included in the revised manuscript (e.g. please see the **line numbers referenced above and the new S3 Figure**).

To rule out this critical alternative explanation, the authors should provide additional control analyses or physiological metrics. For instance, an analysis of pupil diameter could serve as a proxy for arousal levels and cognitive effort, allowing for a comparison across these three key experimental conditions.

As noted by Reviewer 3 below, we have now added further analyses on the distributions of microsaccade directions in the different retinal image stabilization conditions (please see **S3C, D Figure**). This touches on issues related to expectation and arousal.

We have also shown that constraining the direction distributions does not alter the key results (please see the **modified Figure 2D**, and the **modified Figure 5**).

In addition, our manuscript does report reaction times, which are a robust behavioral measure of arousal. We also now report the links to reaction times in the individual monkeys (please see **S8 Figure**).

However, once again, please note that arousal was expected to be similar regardless of retinal image stabilization direction (see the onset of microsaccadic inhibition in the newly added **S3A Figure**), so our results are more related to the control of foveal oculomotor state (as well as the expectation of the constant peripheral stimulus location in a given block). Consistent with this, Fig. 2D showed similar microsaccade-related discharge regardless of retinal image stabilization condition (also see our responses to Reviewer 3 below). Moreover, the mechanism for peripheral visual sensitivity enhancement turned out to be the same as in the control condition (Fig. 5). This makes us believe that arousal was not the sole determinant of our findings. Please see **lines 340-343, 576-579, 606-609, 722-723, 1596-1599**.

Finally, concerning pupil diameter, unfortunately, we did not measure the monkeys' eye movements with a video-based eye tracker for this study. Moreover, we are currently in an unfortunate holding period in our lab, awaiting the renewal of our animal license (a process that is taking much longer than anticipated). This means that it can take many more months before we might be able to even consider starting training monkeys again, and eventually recording some new control sessions with pupil measurements. This could be a nice follow-up study (eventually).

2. The authors adopted a data exclusion strategy based on the presence of microsaccades during the analysis period. However, this approach may introduce a subtle selection bias. Specifically, in the "microsaccade condition," analysis was restricted to trials in which exactly one microsaccade was successfully elicited within a 50-400 ms window following the fixation spot jump; trials with no microsaccades or multiple microsaccades were excluded. This inclusion criterion effectively introduces a selection bias toward trials characterized by high motor readiness and elevated task engagement. Trials in which microsaccades failed to occur—potentially due to attentional fluctuations, slower processing, or insufficient motor drive—were systematically excluded, thereby skewing the sample for the experimental condition toward states of high vigilance and optimal executive performance. Conversely, in the cueing control experiment, the authors excluded trials in which microsaccades occurred, retaining only those with "stable fixation." Although this procedure was intended to eliminate motor confounds, it effectively selected for trials in which subjects successfully implemented active oculomotor inhibition. Consequently, the control condition may inadvertently represent a state of "active suppression."

We have now clarified issues related to this comment, as we summarize below.

Please note that the goal of the fixation spot jump was exactly to trigger a microsaccade with a small foveal error, such that we could inspect peri-microsaccadic intervals. Thus, following the fixation spot jump with an eye movement was the explicit instruction to the subjects, and it was a scientific tool to allow us to measure peri-microsaccadic intervals. In practice, there were rarely ever trials with multiple microsaccades. This is because saccades in response to very small eccentricity jumps generally have high latencies (please see the classic Hallett studies on saccadic latencies; and also Hafed and Goffart, 2020 in monkeys). Similarly, having no microsaccades would mean not having pre- and post-microsaccadic intervals to analyze in the first place, and that is why we had to exclude such trials. We have now clarified this information further in the revised manuscript. For example, please see **lines 595-596, 600-601, 1112-1113, 1181-1186**.

Concerning the converse situation in the control experiment, once again, this was done to remove pre- and post-microsaccadic influences, which was the purpose of the experiment. Please also note that we did not compare performance across experiments. Rather, in each experiment, we compared the same and opposite directions. Thus, even if the cognitive state in the cue control experiment was slightly different from that in the microsaccade experiment, the key is that within each experiment, we attempted to isolate the differential influences (or lack thereof) of pre- and post-microsaccadic intervals. We have now clarified this information further in, for example, **lines 1141-1143**.

3. While the authors' elegantly designed "cueing control experiment" effectively rules out exogenous attention driven by the fixation jump (retinal transient), it requires subjects to engage in active inhibition. Such suppression—exerted to counteract exogenous oculomotor capture—is typically accompanied by a downregulation of visual gain at the target location. Therefore, the absence of an observed sensitivity enhancement in the control experiment may result from the gain provided by exogenous attention being offset by the attenuation induced by active suppression.

The authors are requested to clarify this possibility.

We think that this is unlikely for the following reasons.

First, our peripheral target was spatially dissociated from the foveal cue, and any "oculomotor capture" should happen at the foveal cue and not at the peripheral target. In other words, it is not clear why a foveal cue should capture a large saccade at a much higher eccentricity.

Second, and in a related manner, exogenous attention would be at the foveal cue and not at the peripheral target (we dissociated the two).

Third, the control experiment tested whether a foveal transient, in the absence of microsaccades, could reproduce the differential peripheral directional modulations observed in the main experiment with microsaccades. It did not. Therefore, regardless of whether active inhibition in the fovea influenced overall sensitivity or not, the critical directional effect remained absent without microsaccade generation.

Fourth, for a brief transient, especially a foveal one like we used, not getting a microsaccadic response is actually the expectation not the exception. This is because, as mentioned above, triggering small microsaccades is effortful to the oculomotor system. Also, foveal transients (like peripheral ones) cause inhibition of microsaccades before any capture (please see, for example, Buonocore & Hafed, 2023). So, we actually believe that there was no need for active suppression in our cue control experiment, especially because the foveal stimulus was transient, and because there was no persistent visual stimulus to capture the eye.

All of these points are now addressed in the revised manuscript. For example, please see, among others, **lines 595-596, 600-601, 1112-1113, 1141-1143, 1181-1186**.

Minor Comments:

1. The authors defined the "Same" category using a broad window of ±45 deg. Have the authors analyzed how the modulation index changes if a stricter criterion (e.g., ±22.5 deg) is applied? A narrower window might reveal stronger, more directionally specific effects, or conversely, show that the effect is broadly tuned.

Thank you for this comment, which is also related to one made later by Reviewer 3 below. We have now modified **Fig. 2D** and **Fig. 5** to include both genuine "towards" and genuine "opposite" movements within the "same" and "opposite" conditions, respectively; we did this exactly to show that the effects do indeed depend on microsaccade direction and not just the direction of our induced foveal error (i.e. there could be non-same microsaccades in the same trials, or non-opposite microsaccades in the opposite trials). Indeed, the effects were rendered stronger, especially in Fig. 5 and in the post-saccadic epoch. Note, however, that this analysis was still with +/- 45 deg, in order to make sure that we have sufficient data for the analyses. In our previous work (Chen et al., 2015), we found that a stricter criterion strengthened the effects, so we expect the same here; in fact, all of our three monkeys were used in previous demonstrations of pre-microsaccadic enhancement (Monkey N was in the original Chen et al., 2015 study, and monkeys A and M were in

the Wu & Hafed, 2025 replication) (please see **lines 466-467, 804-806, 1005-1006**). Please also see the responses to Reviewer 3 below.

2. Peak statistics are highly sensitive to noise and outliers in single trials; please clarify the rationale for using the "peak" value as the denominator for normalization.

We have now added this information in **lines 1018-1022**. Briefly, we picked visually-responsive neurons (based on explicit statistical tests), so there was always a robust peak visual burst, and we also took the peak of the average firing rate curve (obtained from many trial repetitions). So, this increased our confidence that our modulation indices were well behaved. This is common practice.

3. In the human psychophysical experiments, the authors estimated perceptual thresholds (defined as the 50% point) by fitting cumulative Gaussian functions using the psignifit toolbox. It is strongly suggested that the authors also quantify and compare the "width" parameters of the psychometric functions across conditions. From Fig. 6d, the width appears to differ among the three conditions.

Thank you. We have now added documentation of the width parameters of the psychometric curves. Please see **lines 617-621, 642-645, 1214-1216**.

Responses to Reviewer 2

This study investigates the relationship between microsaccades and their effects on extrafoveal visual sensitivity and behavior using a novel paradigm that induces microsaccades and systematically manipulates their direction via retinal image stabilization. The results convincingly demonstrate that microsaccades can be experimentally induced and that their directions are strongly dictated by the imposed foveal visual error across four monkeys. Importantly, superior colliculus (SC) foveal neural activity prior to stimulus onset varies as a function of induced microsaccade direction (toward vs. opposite the stimulus), whereas extrafoveal activity is largely independent of microsaccade direction. Moreover, these direction-dependent effects extend into post-stimulus SC activity (in visual-motor neurons) and are linked to behavioral performance in both monkeys and humans. These findings appear to follow the same peri-microsaccadic principles observed in previous work. Overall, the manuscript is very clearly written, the methodological procedures are carefully executed, the analyses are appropriate, and the results are highly interesting with clear interpretations and implications. Together, they provide a significant advancement in our understanding of the causal relationship between microsaccades and attention. I therefore have only several relatively minor comments.

Thank you very much for carefully reviewing our manuscript. We have attempted to fully address all of the reviewer and editor comments in the revised version.

First, the authors convincingly show effective manipulation of microsaccade directions using their paradigm across four monkeys (Fig. S2). However, for many of the other major findings, the consistency of results across individual monkeys is not clearly presented. While it is understandable that some animals may not have sufficient data to produce equally robust effects, it remains important to demonstrate that the key findings are qualitatively consistent across subjects. Providing supplementary analyses or individual-subject plots would strengthen the generalizability of the conclusions.

We have now added **S8 Figure** showing individual monkey results, including the links of the visual bursts to their respective saccadic reaction times, which were consistent with the overall population data.

Please also note that all three monkeys were shown to demonstrate pre-microsaccadic enhancement in the past (in different sensory and experimental contexts): monkey N was used in the original study of Chen et al., 2015; and monkeys A and M were used in the later replication of Wu & Hafed, 2025. Thus, we are confident that our observations hold at the individual monkey level. This is now also mentioned in the revised manuscript (please see **lines 466-467, 804-806**).

Finally, please note that our example neurons in Fig. 3 were from multiple monkeys. Specifically, the first 3 neurons were from monkey N, and the last two neurons were from monkey A. This is now explicitly indicated (please see **lines 397, 423-424**).

Second, the pre-stimulus results show marked differences between foveal and extrafoveal SC neurons (Fig. 2). However, the effect size in foveal neurons appears modest (e.g., $p = 0.0441$ in Fig. 2c). Additionally, control microsaccades were categorized as "towards" and "rest" (Fig. 2d), and the response profiles appear similar between experiment-towards and control-towards conditions (and likewise between opposite and control-rest). This is particularly intriguing, as it suggests that microsaccades, regardless of whether they are experimentally induced or naturally generated, produce comparable neural effects. This finding implies that microsaccade generation per se may be sufficient to modulate foveal activity and influence task-related performance. Furthermore, because there is no warning cue in the control condition (line 329), these results help mitigate potential confounds introduced by the experimental manipulation itself. The authors are encouraged to discuss why the
foveal neuronal differences are relatively small despite statistical significance, and provide a more detailed comparison between experimentally induced and naturally generated microsaccades (especially in Fig. 2b), explicitly testing whether these two classes produce statistically indistinguishable effects on foveal and extrafoveal activity as well as on behavior.

We fully agree that regular microsaccades produce comparable results, and this was one of the key points that we wanted to convey to the readership. So, we are very happy that you agree with us. Indeed, besides our original study (Chen et al., 2015), we have now replicated this phenomenon in (Wu & Hafed, 2025), as well as in this latest manuscript. So, we are confident that this is a robust phenomenon that can be observed across multiple studies.

Concerning the relatively small differences seen in Fig. 2B, C, we have now clarified this in the revised manuscript. Please see **lines 301-304**. Briefly, we think that there are multiple reasons to expect a small difference:

- We used a very subtle manipulation of foveal error, with a magnitude of 0.058-0.128 deg only. When compared to the sizes of foveal RF's in the SC (e.g. Chen et al., 2019; also see Hafed & Krauzlis, 2008), such a shift was smaller than RF size and thus was not expected to dramatically alter the tonic activity of the neurons
- We were measuring saccade-free activity in Fig. 2B, C. Such activity is low-level compared to motor bursts, and its modulations are expectedly smaller. In fact, the effects in Fig. 2C are comparable to (and not much smaller than) those that we obtained earlier (e.g. Fig. 6 of Hafed & Krauzlis, 2008, which had even larger foveal errors than what we had here). Indeed, when we looked at foveal motor bursts (Fig. 2D), the differences between the directions were much clearer

For us, having as subtle an experimental manipulation of foveal state was absolutely critical. For example, we did not want microsaccade amplitudes to be dramatically altered by retinal image stabilization, or inter-microsaccade drifts to become too fast. The key was to achieve differential modulation in the foveal SC with as small a foveal error as possible, which we succeeded in having.

Finally, concerning the final point, please note that Fig. 2B was showing saccade-free epochs. However, in Fig. 2D, we did perform such a comparison. Motor bursts were similar for microsaccades of similar direction, whether the microsaccades occurred in control or retinal image stabilization trials. In the **modified Fig. 2D**, we have now added yet another a curve for microsaccades opposite the RF (please see Reviewer 3 below for more information). This makes this figure exhaustive and also consistent with what to expect from the foveal region of the SC in terms of microsaccade generation (e.g. Hafed et al., 2009; Hafed & Krauzlis, 2012).

We also took the same approach in the **modified Fig. 5**. This makes the figure demonstrate that the modulations that we got were qualitatively similar whether a microsaccade was occurring in the control condition or not. The modified figure also addresses a comment by Reviewer 3 below.

Third, one of the major contributions of this study is the implementation of a paradigm that experimentally manipulates microsaccade occurrence and direction, thereby enabling causal inference regarding their influence on foveal and extrafoveal neural activity as well as behavior. While it is clear that the paradigm successfully induces microsaccades and biases their direction, it is not entirely clear from Fig. 1d or from the description (line 157: "Microsaccade directions were overwhelmingly dictated by the direction of forced foveal visual error") whether microsaccades are reliably evoked within a defined time window (e.g., 300 ms) after foveal retinal image stabilization onset. Does stabilization almost always trigger a microsaccade within this interval, and does this microsaccade reliably occur in the direction of the new fixation target? Or is there still a proportion of trials in which no microsaccade occurs within this time window? Providing probability density functions or cumulative distributions of microsaccade latency and directional bias following stabilization onset would greatly clarify the reliability and temporal dynamics

of the manipulation.

Thank you for highlighting this important missing detail. We have now added this information in the new **S3A, B Figure**. Briefly, the onset of retinal image stabilization is a (subtle) foveal sensory transient, which is expected to alter microsaccade frequency as the oculomotor system balances between completing the preceding motor plan versus reacting to the new sensory transient (e.g. Buonocore and Hafed, 2023). Specifically, we saw a subtle microsaccadic inhibition followed by rebound, indicating that the manipulation did indeed reset microsaccade generation processes, and trigger subsequent microsaccadic responses in the rebound epoch. We had originally focused on only the directions because it is those directions that were relevant for our analyses of differential modulations on sensitivity. However, we now include rate as well. Please also see Tian et al., 2016, 2018 for more details on our foveal retinal image stabilization impacts on microsaccades (please see **lines 254-281**).

Finally, in line 206 ("With large forced visual errors, retinal image stabilization can massively destabilize the oculomotor control system"), the terms "large" and "small" forced visual errors should be quantitatively defined. Providing specific angular values or displacement magnitudes would improve clarity and reproducibility.

We have now clarified this information. Please see **lines 235-240, 1537-1538**. Briefly, we meant that the manipulation eliminates the negative feedback control loop, and reveals the high gain of the saccade generator system in the brainstem.

Responses to Reviewer 3

Reviewer #3: In this study, Hafed & colleagues describe a novel paradigm to experimentally control the direction of fixational microsaccades while measuring the sensitivity to extrafoveal stimuli. They demonstrate convincingly that (a) their gaze-contingent image stabilization paradigm can achieve exquisite control of microsaccade direction and (b) the foveal manipulation modulates foveal SC visuo-motor neuron responses, while extra-foveal neurons are unaffected, and (c) that this manipulation impacts visually driven extrafoveal stimulus detection. These results draw a causal link between foveal oculomotor state and extrafoveal sensitivity, thereby resolving the nature of a long-debated connection between the two.

Thank you very much for the careful review of our paper. We have made a genuine effort to address all reviewer and editor comments.

I have a couple of points that I would like the authors to clarify:

(A) Although not quantified, the distribution of the microsaccade directions in the "same" condition seems to be tighter compared to the other three conditions (Fig 1c). This trend seems to be consistent across all 4 animals (Fig S2a-d). Can the author quantify this asymmetry and comment on the possible reasons behind it? Is it because of expectation, since the location of the extrafoveal stimulus is fixed per

session? If there is indeed such an expectation bias toward the extrafoveal stimulus location, how much does that impact the main conclusions of the study?

Thank you very much for noticing this intriguing observation. We have now quantified this asymmetry. Specifically, please see the new **S3C, D Figure** and **lines 254-281**. The variance in microsaccade directions was indeed smaller in the "same" condition. We also analyzed microsaccade rates during the retinal image stabilization interval in the new **S3B Figure**. Here, we found that there were slightly more microsaccades in the "same" than "opposite" conditions in the rebound period after the earlier subtle microsaccadic inhibition. Rebound microsaccades are the oculomotor responses to the foveal error, after the initial resetting by the transient (e.g. Buonocore & Hafed, 2023), and they are driven by top-down control (e.g. Peel et al., 2016). So, it seems that target expectation does indeed play a role here. We have now clarified the text on this. Please see **lines 254-281**. Therefore, in addition to the effects of target expectation and arousal on saccadic reaction times that we had originally reported, we now have documentation on the microsaccades themselves. Thank you for alerting us to this.

However, and as mentioned for Reviewer 1 above, we do not think that this alters the main conclusions of our study. This is because we still established a differential modulation of oculomotor state and foveal SC activity, leading to differential peripheral sensitivity enhancement. For example, please see **lines 340-343, 576-579, 606-609, 722-723, 1596-1599**. Importantly, the results of Fig. 5 suggest that there were substantial numbers of trials (with post-microsaccadic stimuli) in which visual sensitivity was actually reduced, rather than enhanced, even on "same" trials. Thus, target expectation itself was not the sole source of visual sensitivity modulations that we observed. Also, one of the reasons that we did the human experiment of Fig. 6 was exactly to have a condition without the arousal or target expectation that were inevitable in our neurophysiological experiments (please see **lines 603-609**).

Finally, we have now added another control analysis. Specifically, we have now constrained the angles of microsaccades that we included in the analyses, even in the opposite condition (the **modified Figure 5**). Thus, we matched the ranges of microsaccade directions across conditions. The results were qualitatively the same. This is expected because our prior work (e.g. Chen et al., 2015) showed that a difference of even +/- 22.5 deg from a given microsaccade is sufficient to alter the pre-microsaccadic enhancement. So, if our microsaccades were 180 deg away (even with broader variance in the opposite condition), we should still expect a differential modulation from the same condition.

(B) I am puzzled by the logic and the results of the human psychophysics experiments. The neural and monkey behavioral results largely rest on induced microsaccades that occur before the onset of the extrafoveal stimulus. What is then the basis of the stated expectation of "pre-microsaccadic enhancement of human extrafoveal contrast sensitivity in non-attention tasks" (lines 501-503)? The study finds enhancement for congruent microsaccade direction and suppression for opposite direction if the grating appeared within 50ms before microsaccade onset, and no differential modulation for gratings appearing within 50ms after microsaccade onset. Isn't this the opposite temporal trend to what might be expected from the

neural results?

We apologize for the lack of clarity here. We have now remedied this in the revised manuscript. Please see **lines 569-581, 603-606**.

Briefly, our monkey results revealed that the mechanism through which we obtained peripheral enhancement with retinal image stabilization was still **time-locked** to microsaccade generation (e.g. Fig. 5). Specifically, peripheral enhancement was restricted to the pre-microsaccadic interval. Thus, what our monkey manipulations achieved was to manipulate oculomotor state in order to alter the relative directions between stimulus direction and microsaccade direction, but the revealed mechanism was still pre-microsaccadic enhancement, which we find remarkable (please also see Reviewer 2 above). Thus, in the human experiment, we used a similar manipulation (foveal error) to trigger microsaccades in a given direction, and we checked for pre-microsaccadic modulations in perception. There is no contradiction between the two experiments.

Responses to Academic Editor

It would be appropriate to provide some discussion of the importance of microsaccades to compensate for small head motions in real world vision, for the purposes of retinal stabilization (e.g., Aytekin, Victor, Rucci, J Neurosci, 2014). In that manner, microsaccades and other very small eye movements may serve the purpose of acting like a biological steadicam or such.

Done. Please see **lines 100-103, 120-121**. We agree that this role is consistent with the function of microsaccades in optimizing eye position under a variety of behavioral contexts.
