# Supplementary material for: Microsaccadic eye movement generation is sufficient to enhance peripheral visual sensitivity
Source: PLoS Biol. 2026 Aug 21;24(8):e3003968. doi: 10.1371/journal.pbio.3003968 (PMC13521465; doi:10.1371/journal.pbio.3003968)
Supplement: Peer review history

# Editor report: Decision Letter 0

**DOI:** 10.1371/journal.pbio.3003968.r001

**Authors:** Taylor Hart, PhD

2 Feb 2026

Dear Dr Hafed,

Thank you for submitting your manuscript entitled "Foveal action for the control of extrafoveal vision" for consideration as a Research Article by PLOS Biology.

Your manuscript has now been evaluated by the PLOS Biology editorial staff, as well as by an academic editor with relevant expertise, and I am writing to let you know that we would like to send your submission out for external peer review.

However, before we can send your manuscript to reviewers, we need you to complete your submission by providing the metadata that is required for full assessment. To this end, please login to Editorial Manager where you will find the paper in the 'Submissions Needing Revisions' folder on your homepage. Please click 'Revise Submission' from the Action Links and complete all additional questions in the submission questionnaire.

Once your full submission is complete, your paper will undergo a series of checks in preparation for peer review. After your manuscript has passed the checks it will be sent out for review. To provide the metadata for your submission, please Login to Editorial Manager (https://www.editorialmanager.com/pbiology) within two working days, i.e. by Feb 04 2026 11:59PM.

If your manuscript has been previously peer-reviewed at another journal, PLOS Biology is willing to work with those reviews in order to avoid re-starting the process. Submission of the previous reviews is entirely optional and our ability to use them effectively will depend on the willingness of the previous journal to confirm the content of the reports and share the reviewer identities. Please note that we reserve the right to invite additional reviewers if we consider that additional/independent reviewers are needed, although we aim to avoid this as far as possible. In our experience, working with previous reviews does save time.

If you would like us to consider previous reviewer reports, please edit your cover letter to let us know and include the name of the journal where the work was previously considered and the manuscript ID it was given. In addition, please upload a response to the reviews as a 'Prior Peer Review' file type, which should include the reports in full and a point-by-point reply detailing how you have or plan to address the reviewers' concerns.

During the process of completing your manuscript submission, you will be invited to opt-in to posting your pre-review manuscript as a bioRxiv preprint. Visit http://journals.plos.org/plosbiology/s/preprints for full details. If you consent to posting your current manuscript as a preprint, please upload a single Preprint PDF.

Feel free to email us at plosbiology@plos.org if you have any queries relating to your submission.

Kind regards,

Taylor

Taylor Hart, PhD,

Associate Editor

PLOS Biology

thart@plos.org

# Peer review: Decision Letter 1

**DOI:** 10.1371/journal.pbio.3003968.r002

**Authors:** Taylor Hart, PhD

1 Apr 2026

Dear Dr Hafed,

Thank you for your patience while your manuscript "Foveal action for the control of extrafoveal vision" was peer-reviewed at PLOS Biology. It has now been evaluated by the PLOS Biology editors, an Academic Editor with relevant expertise, and by several independent reviewers.

In light of the reviews, which you will find at the end of this email, we would like to invite you to revise the work to thoroughly address the reviewers' reports.

As you will see, the reviewers expressed interest in the methodology and the findings. However, Reviewer 1 raised some concerns about possible confounds and unaddressed sources of bias, and all reviewers requested additional analyses, clarifications, and discussion points. In your revision, you should thoroughly address all of the reviewers' concerns, including through new analyses, quantifications, and textual changes. You should also address the Academic Editor's additional point (included below the reviews).

Given the extent of revision needed, we cannot make a decision about publication until we have seen the revised manuscript and your response to the reviewers' comments. Your revised manuscript is likely to be sent for further evaluation by all or a subset of the reviewers.

In addition to these revisions, you will need to complete some formatting changes, which you will receive in a follow up email. A member of our team will be in touch with a set of requests shortly.

We expect to receive your revised manuscript within 3 months. Please email us (plosbiology@plos.org) if you have any questions or concerns, or would like to request an extension.

At this stage, your manuscript remains formally under active consideration at our journal; please notify us by email if you do not intend to submit a revision so that we may withdraw it.

**IMPORTANT - SUBMITTING YOUR REVISION**

Your revisions should address the specific points made by each reviewer. Please submit the following files along with your revised manuscript:

1. A 'Response to Reviewers' file - this should detail your responses to the editorial requests, present a point-by-point response to all of the reviewers' comments, and indicate the changes made to the manuscript.

*NOTE: In your point-by-point response to the reviewers, please provide the full context of each review. Do not selectively quote paragraphs or sentences to reply to. The entire set of reviewer comments should be present in full and each specific point should be responded to individually, point by point.

You should also cite any additional relevant literature that has been published since the original submission and mention any additional citations in your response.

2. In addition to a clean copy of the manuscript, please also upload a 'track-changes' version of your manuscript that specifies the edits made. This should be uploaded as a "Revised Article with Changes Highlighted" file type.

*Re-submission Checklist*

When you are ready to resubmit your revised manuscript, please refer to this re-submission checklist: https://plos.io/Biology_Checklist

To submit a revised version of your manuscript, please go to https://www.editorialmanager.com/pbiology/ and log in as an Author. Click the link labelled 'Submissions Needing Revision' where you will find your submission record.

Please make sure to read the following important policies and guidelines while preparing your revision:

*Published Peer Review*

Please note while forming your response, if your article is accepted, you may have the opportunity to make the peer review history publicly available. The record will include editor decision letters (with reviews) and your responses to reviewer comments. If eligible, we will contact you to opt in or out. Please see here for more details:

https://blogs.plos.org/plos/2019/05/plos-journals-now-open-for-published-peer-review/

*PLOS Data Policy*

Please note that as a condition of publication PLOS' data policy (http://journals.plos.org/plosbiology/s/data-availability) requires that you make available all data used to draw the conclusions arrived at in your manuscript. If you have not already done so, you must include any data used in your manuscript either in appropriate repositories, within the body of the manuscript, or as supporting information (N.B. this includes any numerical values that were used to generate graphs, histograms etc.). For an example see here: http://www.plosbiology.org/article/info%3Adoi%2F10.1371%2Fjournal.pbio.1001908#s5

*Blot and Gel Data Policy*

We require the original, uncropped and minimally adjusted images supporting all blot and gel results reported in an article's figures or Supporting Information files. We will require these files before a manuscript can be accepted so please prepare them now, if you have not already uploaded them. Please carefully read our guidelines for how to prepare and upload this data: https://journals.plos.org/plosbiology/s/figures#loc-blot-and-gel-reporting-requirements

*Protocols deposition*

To enhance the reproducibility of your results, we recommend that if applicable you deposit your laboratory protocols in protocols.io, where a protocol can be assigned its own identifier (DOI) such that it can be cited independently in the future. Additionally, PLOS ONE offers an option for publishing peer-reviewed Lab Protocol articles, which describe protocols hosted on protocols.io. Read more information on sharing protocols at https://plos.org/protocols?utm_medium=editorial-email&utm_source=authorletters&utm_campaign=protocols

Thank you again for your submission to our journal. We hope that our editorial process has been constructive thus far, and we welcome your feedback at any time. Please don't hesitate to contact us if you have any questions or comments.

Sincerely,

Taylor

Taylor Hart, PhD,

Associate Editor

PLOS Biology

thart@plos.org

------------------------------------

REVIEWS:

Reviewer #1: Summary of the Manuscript:

This study employs a novel Retinal Image Stabilization (RIS) technique in macaques to manipulate foveal motor planning, concurrently with neurophysiological recordings in the Superior Colliculus (SC), and combines this approach with human psychophysical experiments. The goal is to dissect the causal relationship between microsaccade generation and peripheral visual sensitivity. The authors report that the foveal oculomotor state is sufficient to causally modulate peripheral visual sensitivity and behavioral reaction times in a directionally specific manner (i.e., enhancement in the "Same" direction). Furthermore, the findings suggest this modulation is mediated by an amplification of pre-microsaccadic gain and an attenuation of post-microsaccadic suppression.

While the topic is novel and the combined RIS-electrophysiology methodology is highly innovative—offering a unique perspective on the long-standing debate regarding causality between microsaccades and attention—some concerns remain regarding the internal validity of the experimental design, particularly within the human experiments, and the control of confounding variables introduced by the RIS manipulation, such as cognitive conflict and arousal dynamics.

Major Comments:

1. The authors acknowledge two critical side effects of the RIS paradigm. First, the "fighting the drift" phenomenon, in which monkeys actively counteract the retinal drift error to maintain fixation. This implies that, particularly in the "Opposite" condition, the animals likely experience a state of elevated cognitive load, characterized by a conflict between the internally generated "motor plan" and the external "task goal" of maintaining fixation. Second, the "temporal cueing" effect, whereby the onset of the RIS itself acts as an unspecific warning signal. This latter effect explains why reaction times were slowest in the Control condition, where no directional plan is introduced.

These facts collectively suggest that the RIS manipulation does not merely isolate the "oculomotor plan" in a pure form but likely induces significant changes in the animal's global cognitive and physiological state. Consequently, the psychological and physiological contexts of the "Same," "Opposite," and "Control" conditions are not equivalent. Recent frameworks in neurodynamics (e.g., Arousal as a universal embedding for spatiotemporal brain dynamics, Nature, 2025) suggest that arousal operates not as a simple linear gain but across a complex, high-dimensional manifold. If the RIS manipulation places the "Same" and "Opposite" conditions at distinct coordinates on this arousal/cognitive state manifold, then the observed differences in visual sensitivity could be a downstream consequence of these global state alterations, rather than a direct, specific causal result of the microsaccade plan per se.

To rule out this critical alternative explanation, the authors should provide additional control analyses or physiological metrics. For instance, an analysis of pupil diameter could serve as a proxy for arousal levels and cognitive effort, allowing for a comparison across these three key experimental conditions.

2. The authors adopted a data exclusion strategy based on the presence of microsaccades during the analysis period. However, this approach may introduce a subtle selection bias. Specifically, in the "microsaccade condition," analysis was restricted to trials in which exactly one microsaccade was successfully elicited within a 50-400 ms window following the fixation spot jump; trials with no microsaccades or multiple microsaccades were excluded. This inclusion criterion effectively introduces a selection bias toward trials characterized by high motor readiness and elevated task engagement. Trials in which microsaccades failed to occur—potentially due to attentional fluctuations, slower processing, or insufficient motor drive—were systematically excluded, thereby skewing the sample for the experimental condition toward states of high vigilance and optimal executive performance. Conversely, in the cueing control experiment, the authors excluded trials in which microsaccades occurred, retaining only those with "stable fixation." Although this procedure was intended to eliminate motor confounds, it effectively selected for trials in which subjects successfully implemented active oculomotor inhibition. Consequently, the control condition may inadvertently represent a state of "active suppression."

3. While the authors' elegantly designed "cueing control experiment" effectively rules out exogenous attention driven by the fixation jump (retinal transient), it requires subjects to engage in active inhibition. Such suppression—exerted to counteract exogenous oculomotor capture—is typically accompanied by a downregulation of visual gain at the target location. Therefore, the absence of an observed sensitivity enhancement in the control experiment may result from the gain provided by exogenous attention being offset by the attenuation induced by active suppression. The authors are requested to clarify this possibility.

Minor Comments:

1. The authors defined the "Same" category using a broad window of ±45 deg. Have the authors analyzed how the modulation index changes if a stricter criterion (e.g., ±22.5 deg) is applied? A narrower window might reveal stronger, more directionally specific effects, or conversely, show that the effect is broadly tuned.

2. Peak statistics are highly sensitive to noise and outliers in single trials; please clarify the rationale for using the "peak" value as the denominator for normalization.

3. In the human psychophysical experiments, the authors estimated perceptual thresholds (defined as the 50% point) by fitting cumulative Gaussian functions using the psignifit toolbox. It is strongly suggested that the authors also quantify and compare the "width" parameters of the psychometric functions across conditions. From Fig. 6d, the width appears to differ among the three conditions.

Reviewer #2: This study investigates the relationship between microsaccades and their effects on extrafoveal visual sensitivity and behavior using a novel paradigm that induces microsaccades and systematically manipulates their direction via retinal image stabilization. The results convincingly demonstrate that microsaccades can be experimentally induced and that their directions are strongly dictated by the imposed foveal visual error across four monkeys. Importantly, superior colliculus (SC) foveal neural activity prior to stimulus onset varies as a function of induced microsaccade direction (toward vs. opposite the stimulus), whereas extrafoveal activity is largely independent of microsaccade direction. Moreover, these direction-dependent effects extend into post-stimulus SC activity (in visual-motor neurons) and are linked to behavioral performance in both monkeys and humans. These findings appear to follow the same peri-microsaccadic principles observed in previous work. Overall, the manuscript is very clearly written, the methodological procedures are carefully executed, the analyses are appropriate, and the results are highly interesting with clear interpretations and implications. Together, they provide a significant advancement in our understanding of the causal relationship between microsaccades and attention. I therefore have only several relatively minor comments.

First, the authors convincingly show effective manipulation of microsaccade directions using their paradigm across four monkeys (Fig. S2). However, for many of the other major findings, the consistency of results across individual monkeys is not clearly presented. While it is understandable that some animals may not have sufficient data to produce equally robust effects, it remains important to demonstrate that the key findings are qualitatively consistent across subjects. Providing supplementary analyses or individual-subject plots would strengthen the generalizability of the conclusions.

Second, the pre-stimulus results show marked differences between foveal and extrafoveal SC neurons (Fig. 2). However, the effect size in foveal neurons appears modest (e.g., p = 0.0441 in Fig. 2c). Additionally, control microsaccades were categorized as "towards" and "rest" (Fig. 2d), and the response profiles appear similar between experiment-towards and control-towards conditions (and likewise between opposite and control-rest). This is particularly intriguing, as it suggests that microsaccades, regardless of whether they are experimentally induced or naturally generated, produce comparable neural effects. This finding implies that microsaccade generation per se may be sufficient to modulate foveal activity and influence task-related performance. Furthermore, because there is no warning cue in the control condition (line 329), these results help mitigate potential confounds introduced by the experimental manipulation itself. The authors are encouraged to discuss why the foveal neuronal differences are relatively small despite statistical significance, and provide a more detailed comparison between experimentally induced and naturally generated microsaccades (especially in Fig. 2b), explicitly testing whether these two classes produce statistically indistinguishable effects on foveal and extrafoveal activity as well as on behavior.

Third, one of the major contributions of this study is the implementation of a paradigm that experimentally manipulates microsaccade occurrence and direction, thereby enabling causal inference regarding their influence on foveal and extrafoveal neural activity as well as behavior. While it is clear that the paradigm successfully induces microsaccades and biases their direction, it is not entirely clear from Fig. 1d or from the description (line 157: "Microsaccade directions were overwhelmingly dictated by the direction of forced foveal visual error") whether microsaccades are reliably evoked within a defined time window (e.g., 300 ms) after foveal retinal image stabilization onset. Does stabilization almost always trigger a microsaccade within this interval, and does this microsaccade reliably occur in the direction of the new fixation target? Or is there still a proportion of trials in which no microsaccade occurs within this time window? Providing probability density functions or cumulative distributions of microsaccade latency and directional bias following stabilization onset would greatly clarify the reliability and temporal dynamics of the manipulation.

Finally, in line 206 ("With large forced visual errors, retinal image stabilization can massively destabilize the oculomotor control system"), the terms "large" and "small" forced visual errors should be quantitatively defined. Providing specific angular values or displacement magnitudes would improve clarity and reproducibility.

Reviewer #3: In this study, Hafed & colleagues describe a novel paradigm to experimentally control the direction of fixational microsaccades while measuring the sensitivity to extrafoveal stimuli. They demonstrate convincingly that (a) their gaze-contingent image stabilization paradigm can achieve exquisite control of microsaccade direction and (b) the foveal manipulation modulates foveal SC visuo-motor neuron responses, while extra-foveal neurons are unaffected, and (c) that this manipulation impacts visually driven extrafoveal stimulus detection. These results draw a causal link between foveal oculomotor state and extrafoveal sensitivity, thereby resolving the nature of a long-debated connection between the two.

I have a couple of points that I would like the authors to clarify:

(A) Although not quantified, the distribution of the microsaccade directions in the "same" condition seems to be tighter compared to the other three conditions (Fig 1c). This trend seems to be consistent across all 4 animals (Fig S2a-d). Can the author quantify this asymmetry and comment on the possible reasons behind it? Is it because of expectation, since the location of the extrafoveal stimulus is fixed per session? If there is indeed such an expectation bias toward the extrafoveal stimulus location, how much does that impact the main conclusions of the study?

(B) I am puzzled by the logic and the results of the human psychophysics experiments. The neural and monkey behavioral results largely rest on induced microsaccades that occur before the onset of the extrafoveal stimulus. What is then the basis of the stated expectation of "pre-microsaccadic enhancement of human extrafoveal contrast sensitivity in non-attention tasks" (lines 501-503)? The study finds enhancement for congruent microsaccade direction and suppression for opposite direction if the grating appeared within 50ms before microsaccade onset, and no differential modulation for gratings appearing within 50ms after microsaccade onset. Isn't this the opposite temporal trend to what might be expected from the neural results?

COMMENTS FROM THE ACADEMIC EDITOR [lightly edited]:

It would be appropriate to provide some discussion of the importance of microsaccades to compensate for small head motions in real world vision, for the purposes of retinal stabilization (e.g., Aytekin, Victor, Rucci, J Neurosci, 2014). In that manner, microsaccades and other very small eye movements may serve the purpose of acting like a biological steadicam or such.

# Author comment: Author response to Decision Letter 2

**DOI:** 10.1371/journal.pbio.3003968.r003

24 Jun 2026

Attachment Submitted filename: Tong_RESPONSES.pdf

# Peer review: Decision Letter 2

**DOI:** 10.1371/journal.pbio.3003968.r004

**Authors:** Taylor Hart, PhD

3 Aug 2026

Dear Dr Hafed,

Thank you for your patience while we considered your revised manuscript "Foveal action for the control of extrafoveal vision" for publication as a Research Article at PLOS Biology. This revised version of your manuscript has been evaluated by the PLOS Biology editors, the Academic Editor and the original reviewers.

Based on the reviews, we are likely to accept this manuscript for publication. All reviewers chose to disclose their identities. Please also make sure to address the following data and other policy-related requests.

IMPORTANT: Please ensure that your next revision addresses the following requirements:

-------------------------

**Title:

We would like to add some more description to the title somewhat to make it more accessible to a broad audience. Is the following alternative acceptable to you?

'Microsaccadic eye movements modify peripheral visual sensitivity'

**Ethics:

Thank you for providing ethical approval information regarding the animal and human experiments. Our policy requires that all ethics information be found in the first subheading of the Materials & Methods section, titled "Ethics Statement". Please move the relevant information (about regulations, approvals, consent) to this new subheading.

In addition, please ensure that you include the national or international regulations to which your animal research adhered (not just institutional guidelines), and the full names of all relevant approval bodies.

**Data:

I see that your Data Availability Statement indicates that data tables are provided, but I could not find these items among the supplementary materials (apologies if I have missed them somewhere).

Please ensure that you make the data underlying the figures available. Our policy requires that the numerical values be provided in a supplementary excel file ("S1 Data" / "S1_Data.xlsx") or as a permanent DOI'd deposition, for the following figure panels:

2H

4E

5

S3D

S6AB

S8JKL

S9

S12D

Please also cite the location of the data clearly in all relevant main and supplementary Figure legends, e.g. “The data underlying this Figure can be found in S1 Data” or “The data underlying this Figure can be found in https://doi.org/10.5281/zenodo.XXXXX”

**Code availability:

Per journal policy, if you have generated any custom code or scripts during the course of this investigation, we require that you make it available without restrictions. Please ensure that the code is sufficiently well documented and reusable, and that your Data Statement in the Editorial Manager submission system accurately describes where your code can be found. Please also ensure that you choose a license for your code and include a Readme file.

Please note that we cannot accept sole deposition of code in GitHub, as this could be changed after publication. However, you can archive this version of your publicly available GitHub code to Zenodo. Once you do this, it will generate a DOI number, which you will need to provide in the Data Accessibility Statement (you are welcome to also provide the GitHub access information). See the process for doing this here: https://docs.github.com/en/repositories/archiving-a-github-repository/referencing-and-citing-content

**Species in abstract:

Please note that per journal policy, the model systems/species studied should be clearly stated in the abstract of your manuscript.

-------------------------

As you address these items, please take this last chance to review your reference list to ensure that it is complete and correct. If you have cited papers that have been retracted, please include the rationale for doing so in the manuscript text, or remove these references and replace them with relevant current references. Any changes to the reference list should be mentioned in the cover letter that accompanies your revised manuscript.

In addition to these revisions, you may need to complete some formatting changes, which you will receive in a follow up email. A member of our team will be in touch with a set of requests shortly. If you do not receive a separate email within a few days, please assume that checks have been completed, and no additional changes are required.

We expect to receive your revised manuscript within two weeks.

To submit your revision, please go to https://www.editorialmanager.com/pbiology/ and log in as an Author. Click the link labelled 'Submissions Needing Revision' to find your submission record. Your revised submission must include the following:

- a cover letter that should detail your responses to any editorial requests, if applicable, and whether changes have been made to the reference list

- a Response to Reviewers file that provides a detailed response to the reviewers' comments (if applicable, if not applicable please do not delete your existing 'Response to Reviewers' file.)

- a track-changes file indicating any changes that you have made to the manuscript.

NOTE: If Supporting Information files are included with your article, note that these are not copyedited and will be published as they are submitted. Please ensure that these files are legible and of high quality (at least 300 dpi) in an easily accessible file format. For this reason, please be aware that any references listed in an SI file will not be indexed. For more information, see our Supporting Information guidelines:

https://journals.plos.org/plosbiology/s/supporting-information

*Published Peer Review History*

Please note that you may have the opportunity to make the peer review history publicly available. The record will include editor decision letters (with reviews) and your responses to reviewer comments. If eligible, we will contact you to opt in or out. Please see here for more details:

https://plos.org/published-peer-review-history/

*Press*

Should you, your institution's press office or the journal office choose to press release your paper, please ensure you have opted out of Early Article Posting on the submission form. We ask that you notify us as soon as possible if you or your institution is planning to press release the article.

*Protocols deposition*

To enhance the reproducibility of your results, we recommend that if applicable you deposit your laboratory protocols in protocols.io, where a protocol can be assigned its own identifier (DOI) such that it can be cited independently in the future. Additionally, PLOS ONE offers an option for publishing peer-reviewed Lab Protocol articles, which describe protocols hosted on protocols.io. Read more information on sharing protocols at https://plos.org/protocols?utm_medium=editorial-email&utm_source=authorletters&utm_campaign=protocols

Please do not hesitate to contact me should you have any questions.

Sincerely,

Taylor

Taylor Hart, PhD,

Associate Editor

thart@plos.org

PLOS Biology

Reviewer remarks:

Reviewer #1 [Jianrong Jia]: The authors have adequately addressed all of my concerns. I have no further questions and recommend that this article be published in PLOS Biology.

Jianrong Jia

Reviewer #2 [Chin-An Wang]: The authors have adequately addressed all of my previous concerns. I have no further comments.

Reviewer #3 [Anirvan Nandy]: The authors have done an excellent job of addressing all the points that were raised in the first round of review. The revised manuscript is much improved, and I have no further comments.

# Author comment: Author response to Decision Letter 3

**DOI:** 10.1371/journal.pbio.3003968.r005

10 Aug 2026

Attachment Submitted filename: Tong_RESPONSES_auresp_3.pdf

# Editor report: Decision Letter 3

**DOI:** 10.1371/journal.pbio.3003968.r006

**Authors:** Taylor Hart, PhD

11 Aug 2026

Dear Dr Hafed,

Thank you for the submission of your revised Research Article "Microsaccadic eye movement generation is sufficient to enhance peripheral visual sensitivity" for publication in PLOS Biology. On behalf of my colleagues and the Academic Editor, Frank Tong, I am pleased to say that we can in principle accept your manuscript for publication, provided you address any remaining formatting and reporting issues. These will be detailed in an email you should receive within 2-3 business days from our colleagues in the journal operations team; no action is required from you until then. Please note that we will not be able to formally accept your manuscript and schedule it for publication until you have completed any requested changes.

Please take a minute to log into Editorial Manager at http://www.editorialmanager.com/pbiology/, click the "Update My Information" link at the top of the page, and update your user information to ensure an efficient production process.

PRESS

We frequently collaborate with press offices. If your institution or institutions have a press office, please notify them about your upcoming paper at this point, to enable them to help maximise its impact. If the press office is planning to promote your findings, we would be grateful if they could coordinate with biologypress@plos.org. If you have previously opted in to the early version process, we ask that you notify us immediately of any press plans so that we may opt out on your behalf.

We also ask that you take this opportunity to read our Embargo Policy regarding the discussion, promotion and media coverage of work that is yet to be published by PLOS. As your manuscript is not yet published, it is bound by the conditions of our Embargo Policy. Please be aware that this policy is in place both to ensure that any press coverage of your article is fully substantiated and to provide a direct link between such coverage and the published work. For full details of our Embargo Policy, please visit http://www.plos.org/about/media-inquiries/embargo-policy/.

Thank you again for choosing PLOS Biology for publication and supporting Open Access publishing. We look forward to publishing your study.

Sincerely,

Taylor

Taylor Hart, PhD,

Associate Editor

PLOS Biology

thart@plos.org
